# Motorbike Accidents Involving Delivery Personnel at King Hamad University Hospital, Kingdom of Bahrain: A Retrospective Study

**DOI:** 10.7759/cureus.77778

**Published:** 2025-01-21

**Authors:** Mohammed A Ali, Mohammad A Sajid, Shayma M Ali, Syed R Jilani, Ahsan J Butt, Rommel R Acunin

**Affiliations:** 1 Orthopedic Surgery, King Hamad University Hospital, Busaiteen, BHR; 2 Orthopedic Surgery, Royal College of Surgeons in Ireland - Bahrain, Busaiteen, BHR; 3 Orthopedics and Traumatology, King Hamad University Hospital, Busaiteen, BHR; 4 Biostatistics, Salmaniya Medical Complex, Manama, BHR

**Keywords:** bahrain, food delivery personnel, motorcycle accidents, road safety, road traffic injuries

## Abstract

Background

Motorcycles, favored for commercial commuting, offer the dual benefits of optimizing road system utilization and reducing environmental impact. However, the surge in home delivery services in Bahrain has led to an increase in motorcycle-related injuries. This study aimed to explore the effects of motorcycle accidents on delivery personnel, focusing on injury patterns, severity, and contributing factors.

Methods

This retrospective observational study analyzed 313 cases of delivery personnel involved in motorcycle accidents during their working hours at King Hamad University Hospital, Bahrain, from January 2016 to December 2019. Participants included delivery personnel aged 18-60 years with acute injuries from motorbike accidents who presented within 72 hours. Eligibility required complete medical records and employment verification. Cases involving incomplete data, fatalities before arrival, or accidents with other vehicles were excluded. Data sources included emergency department logs, hospital admission records, operative documents, and standardized forms. Variables included demographics, ambulance use, injury type and location, and interventions such as the need for CT scans, endotracheal intubation, blood transfusion, and trauma code activation. Group comparisons utilized chi-square test, Fisher’s exact test, and Mann-Whitney U tests with significance set at p < 0.05. Subgroup analyses explored associations by age, injury type, and trauma code activation.

Results

The mean age of the patients was 30.1 ± 8.26 years, with more than half (N = 167, 53.4%) aged 30 years old or younger. Of all patients, 25 (8%) had trauma code activation, and 175 (55.9%) utilized an ambulance. The most common location of injury was the lower limb (N = 205, 65.5%), followed by the upper limb (N = 164, 52.4%) and the head (N = 64, 20.4%). The median (range) of hospital stay days was 1 (1-32) days. Abrasions were the major type of injury sustained by the patients (N = 233, 74.4%), followed by lacerations (N = 45, 14.4%). Ambulance use was significantly higher in patients with trauma code activation (p = 0.001), head injury (p = 0.042), and pelvic injury (p = 0.047). Blood transfusion was significantly higher among those with abdominal injury (p = 0.002). There were no fatalities recorded during the study period.

Conclusions

This study highlights the risks faced by young delivery personnel in Bahrain, with lower limb injuries and head trauma being prevalent. Despite the effectiveness of current trauma care protocols in managing injuries, gaps remain in preventing severe injuries. The research emphasizes the need for region-specific measures, such as mandatory helmet laws, the use of protective gear, and the establishment of safer routes for delivery personnel. Furthermore, local awareness campaigns about safe riding practices and enhanced training programs for delivery staff can play a crucial role in reducing injury risks. By providing unique data on injury patterns and trauma care in the Gulf region, the study contributes to improving road safety and trauma management while supporting further research and policy development tailored to the specific needs of delivery personnel in Bahrain.

## Introduction

Motorbikes are increasingly becoming a favored mode of transportation for commercial commuting [1], with their adoption offering dual positive impacts. Firstly, the transition from automobiles to two-wheelers has the potential to optimize road system utilization, thereby enhancing network capacity [2].

Secondly, using two-wheelers presents an opportunity to mitigate environmental impact, given their marginal carbon dioxide contribution to the overall transport sector [2]. However, recent times have witnessed a surge in home delivery services in Bahrain [3], leading to an escalated reliance on motorcycles for efficient food delivery [4]. Unfortunately, this shift has inadvertently increased motorcycle-related injuries [5]. This trend is not exclusive to Bahrain but has also been observed globally with expectations of its persistence and evolution [6]. Global studies reveal distinct injury patterns among young and elderly riders and show how the demographics of motorcycle-related injuries are changing, providing a clearer understanding of the dynamics of motorcycle accidents.

In the broader context of road safety in Bahrain, specific challenges posed by road traffic accidents (RTAs), especially among young individuals, must be acknowledged. A study spanning from 2003 to 2010 highlighted the growing public health problem of RTAs in Bahrain, focusing on fatalities among those aged less than 25 years. Notably, the study revealed a high proportion of young males killed in RTAs emphasizing the urgency for targeted interventions. As our research focuses on motorcycle accidents among delivery personnel in Bahrain, insights into the prevalence of RTAs, especially among the young, provide valuable context. Given Bahrain’s healthcare system's pivotal role, with 17 accredited hospitals and 24 medical centers by the National Health Regulatory Authority, the research aims to contribute vital insights to enhance care [7]. This stresses the critical need for comprehensive and targeted road safety measures, not only for the general population but also for specific demographic groups, such as young males, disproportionately affected by these incidents [7,8].

The primary aim of this study was to investigate the impact of motorcycle accidents on delivery personnel in Bahrain, focusing on injury patterns, severity, and contributing factors. The study aligns with global initiatives to reduce the impact of road traffic injuries, advocating for targeted safety measures and actions specifically for motorcycle delivery personnel.

## Materials and methods

Study design and settings

This retrospective observational study examined 313 cases of delivery personnel involved in motorcycle accidents during working hours. The study was conducted at King Hamad University Hospital (KHUH), Busaiteen, Bahrain, between January 2016 and December 2019. Initial ethical approval was granted by the Research and Ethics Committee of KHUH on March 27, 2018 (approval number KHUH/Research/No. 212/2018). To ensure the continuation of the study, a formal request for an extension was submitted and approved on December 26, 2024, allowing for the completion of the manuscript (approval number RMS-KHUH/IRB/2024-889).

Inclusion and exclusion criteria

Participants were delivery personnel aged 18-60 years who experienced motorbike accidents and were treated at KHUH. Eligible cases involved acute injuries directly related to motorbike accidents. Only patients presenting to the emergency department within 72 hours of the accident and using standard two-wheeled motorbikes associated with delivery services were included. Employment as delivery personnel was verified through occupational details in medical records or self-report. Detailed medical records documenting demographic data, injury type and location, and outcomes were required. Helmet use at the time of the accident was also considered.

Cases were excluded if they involved incomplete medical records, fatalities before hospital arrival, or presentation to KHUH more than 72 hours post-accident. Riders under the influence of drugs or alcohol at the time of the accident, those with non-trauma diagnoses, or preexisting medical conditions unrelated to the accident were excluded. Accidents involving vehicles other than motorbikes or those treated at other facilities before KHUH were also omitted.

Data collection

Data was collected from multiple sources to ensure a comprehensive understanding of each case. These sources included emergency department records, hospital admission logs, operative records, and standardized electronic forms. Emergency department records provided details on initial clinical assessments and immediate interventions. Hospital admission logs documented inpatient management, including admission status and length of stay. Operative records captured data on whether the patient required surgical intervention. Standardized electronic forms were used to consistently document patient information across all cases.

The variables analyzed included demographic details such as age. Pre-hospital factors comprised ambulance utilization, categorized to understand access to emergency care. Injury characteristics were classified by anatomical location, including head, chest, abdomen, pelvis, upper limbs, lower limbs, and spine/back. Specific injuries, such as abrasions, lacerations, and fractures, were detailed for each region. Critical interventions included trauma code activation, CT scans, endotracheal intubation, and blood transfusions.

Statistical analysis

Data analysis was performed using IBM SPSS Statistics for Windows, Version 26.0 (Released 2019; IBM Corp., Armonk, NY, USA). Descriptive statistics summarized continuous variables as mean and SD or median (min-max) and categorical variables as frequencies and percentages. Group comparisons employed chi-square test, Fisher’s exact test, or Mann-Whitney U test, as appropriate. A p-value <0.05 was considered statistically significant. Subgroup analyses explored associations by age, injury type, and trauma code activation.

## Results

The study included 313 individuals involved in motorcycle accidents. Of these, the majority (167 participants, 53.4%) were aged 30 years or younger, with a mean age of 30.1 ± 8.26 years. Hospital admissions were recorded in 64 patients (20.4%), while trauma activation codes were initiated for 25 patients (8%). Ambulance services were utilized by 175 patients (55.9%). Among the cohort, six patients (1.9%) required intubation, and four patients (1.3%) received blood transfusion. Additionally, 34 patients (10.9%) underwent CT imaging, and 36 patients (11.5%) needed surgical intervention. The median hospital stay was one day, ranging from one to 32 days (Table 1).

**Table 1:** Clinicodemographic characteristics of the study patients (N = 313)

Variable	N (%)
Age in years (mean ± SD)	30.1 ± 8.26
≤30 years	167 (53.4)
>30 years	146 (46.6)
Hospital admission	
Yes	64 (20.4)
No	249 (79.6)
Trauma activation code	
Yes	25 (8.0)
No	288 (92.0)
Ambulance employed	
Yes	175 (55.9)
No	138 (44.1)
Endotracheal intubation	
Yes	6 (1.9)
No	307 (98.1)
Blood transfusion	
Yes	4 (1.3)
No	309 (98.7)
CT scans	
Yes	34 (10.9)
No	279 (89.1)
Surgical treatment	
Yes	36 (11.5)
No	277 (88.5)
Length of hospital stay in days, median (min-max)	1.00 (1.00-32.0)

**Figure 1:**
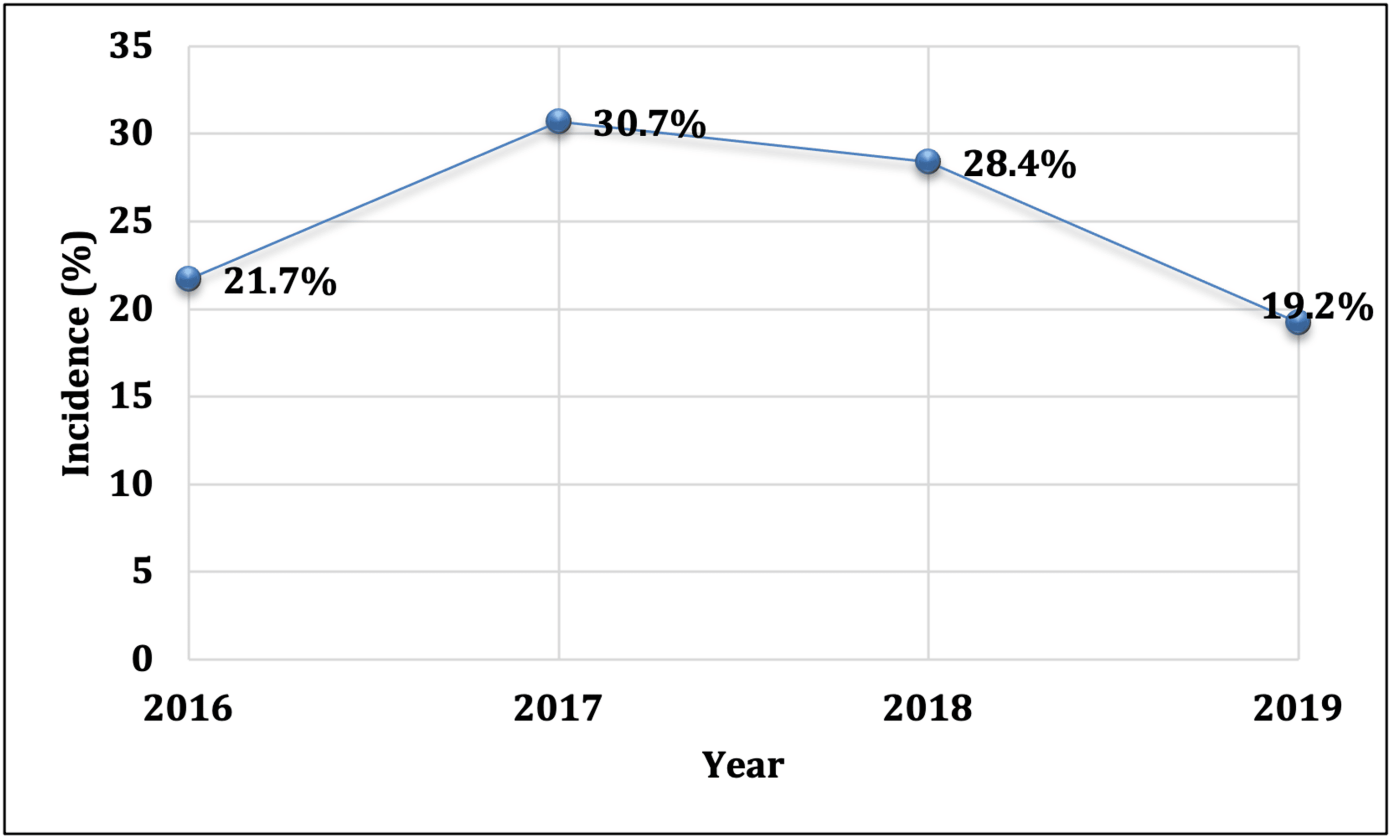
Incidence of motorcycle-related injuries in four years (year 2016-2019)

Figure 1 displays the trauma motorcycle injury trend from 2016 to 2019. The incidence of motorcycle-related injuries was highest in 2017 and lowest in 2019.

**Figure 2:**
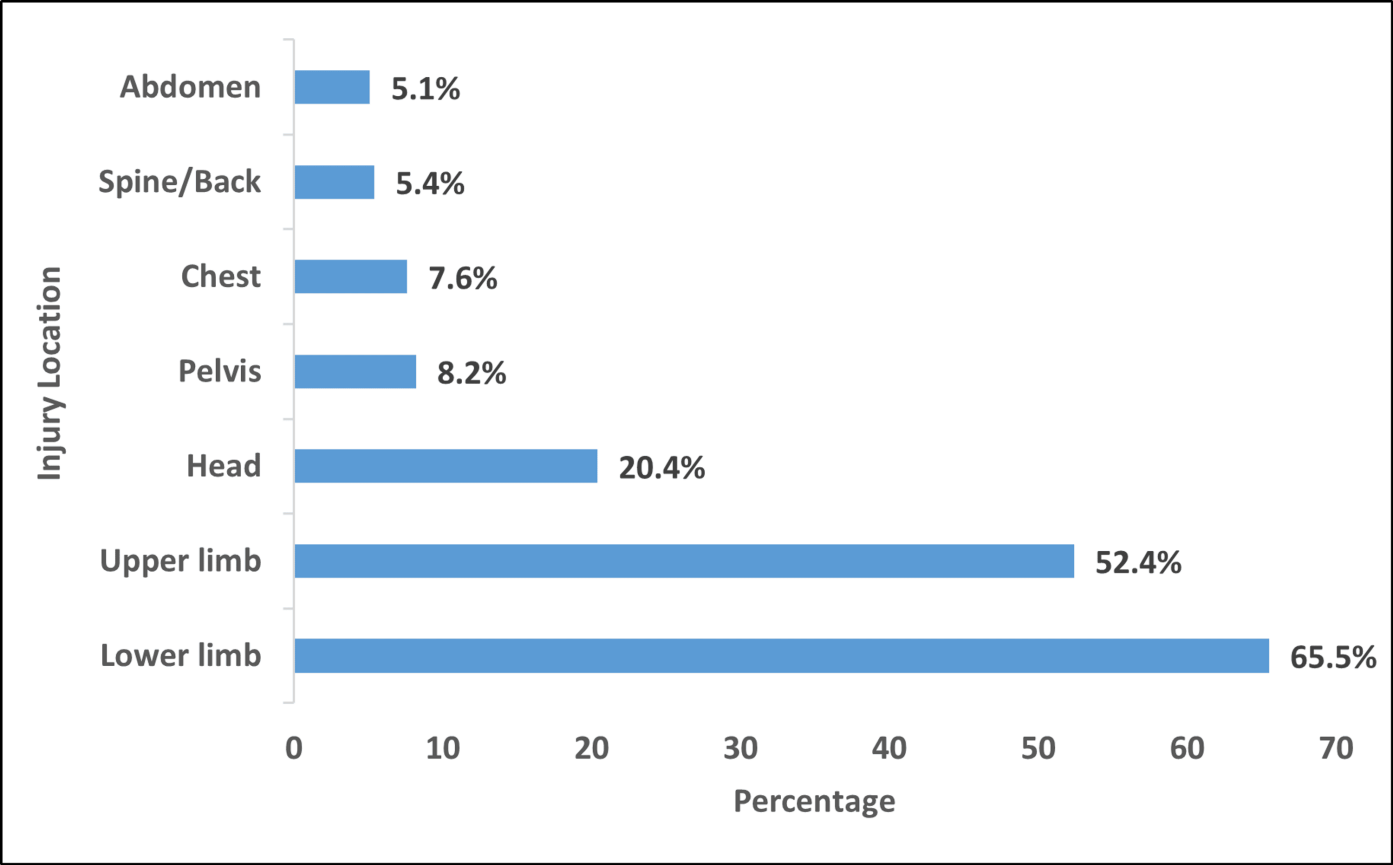
Distribution of injury locations among the study patients (N = 313)

**Figure 3:**
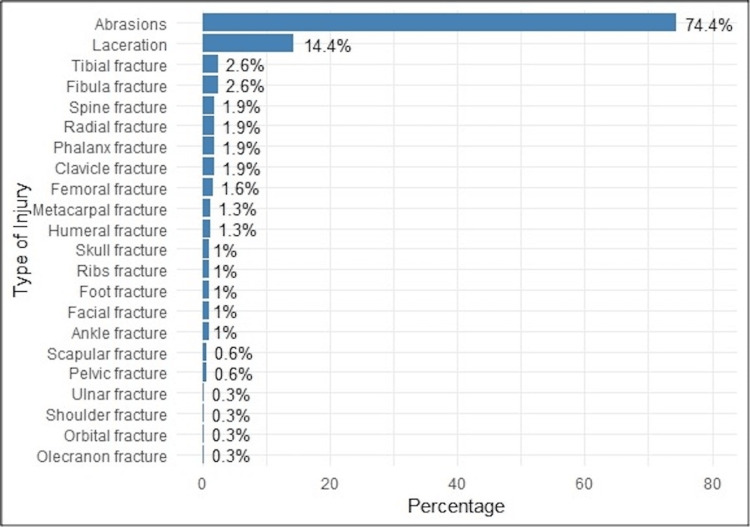
Types of injuries sustained by the study patients (N = 313)

Patients’ data indicated that the lower limb was the most frequently injured region (205 patients, 65.5%), followed by the upper limb (164 patients, 52.4%). Injuries to the head and pelvic regions were observed in 64 patients (20.4%) and 26 patients (8.2%), respectively. Less common injury locations included the chest, spine/back, and abdomen, each comprising less than 10% of cases (Figure 2). Abrasions were the predominant injury type, affecting 233 patients (74.4%), followed by lacerations in 45 patients (14.4%). The least frequent injuries included fractures of the shoulder, orbital, olecranon, and ulnar regions (Figure 3). Many patients sustained multiple injuries involving different types and locations.

Analysis of patients’ characteristics based on their age demonstrated that younger patients (<30 years) had a significantly higher incidence of motorcycle injuries during the years 2017 and 2018, while older patients (>30 years) had a higher incidence in 2016 and 2019 (p = 0.020). Additionally, the younger age group had a statistically significantly higher incidence of upper limb injuries compared with the older age group (p = 0.031) (Table 2).

**Table 2:** Distribution of patients’ characteristics (N = 313) according to age group ^† ^Some patients had multiple types and locations of injury. ^*^ Significant at p <0.05 level (chi-square test) ^**^ Mann-Whitney U test

Characteristic	Age group		p-value
	≤30 years (N = 167)	>30 years (N = 146)	
Hospital admission, N (%)			
Yes	30 (18.0)	34 (23.3)	0.244
No	137 (82.0)	112 (76.7)	
Trauma activation code, N (%)			
Yes	12 (07.2)	13 (08.9)	0.576
No	155 (92.8)	133 (91.1)	
Ambulance employed, N (%)			
Yes	92 (55.1)	83 (56.8)	0.754
No	75 (44.9)	63 (43.2)	
Endotracheal intubation, N (%)			
Yes	02 (01.2)	04 (02.7)	0.321
No	165 (98.8)	142 (97.3)	
Blood transfusion, N (%)			
Yes	02 (01.2)	04 (02.7)	1.000
No	165 (98.8)	142 (97.3)	
Year of injury, N (%)			
2016	30 (18.0)	38 (26.0)	0.020^*^
2017	56 (33.5)	40 (27.4)	
2018	56 (33.5)	33 (22.6)	
2019	25 (15.0)	35 (24.0)	
CT scan, N (%)			
Yes	17 (10.2)	17 (11.6)	0.678
No	150 (89.8)	129 (88.4)	
Surgical treatment, N (%)			
Yes	16 (09.6)	20 (13.7)	0.255
No	150 (89.8)	126 (86.3)	
Injury type^†^, N (%)			
Abrasions	126 (75.4)	107 (73.3)	0.662
Lacerations	21 (12.6)	24 (16.4)	0.331
Others	29 (17.4)	26 (17.8)	0.918
Injury location^†^, N (%)			
Head	34 (20.4)	30 (20.5)	0.967
Abdomen	10 (06.0)	06 (04.1)	0.452
Chest	10 (06.0)	12 (08.2)	0.441
Pelvis	16 (09.6)	11 (07.5)	0.520
Upper limb	97 (58.1)	67 (45.9)	0.031^*^
Lower limb	114 (68.3)	91 (62.3)	0.271
Spine/back	08 (04.8)	09 (06.2)	0.593
Length of hospital stay in days, median (min-max)	1.00 (1.00-32.0)	1.00 (1.00-21.0)	0.131^**^

Surgical treatment was significantly associated with hospital admission, with 33 admitted patients (51.6%) undergoing surgical procedures compared to three non-admitted patients (1.2%, p < 0.001). For injury type, abrasions were more frequent among non-admitted patients (202, 81.1%) compared to admitted patients (31, 48.4%, p < 0.001). Lacerations were observed more commonly among admitted patients (17, 26.6%) than non-admitted patients (28, 11.2%, p = 0.002). Other injury types were significantly higher in admitted patients (30, 46.9%) than in non-admitted patients (25, 10.0%, p < 0.001). Analysis of injury locations showed significant differences for head injuries, which were more common among admitted patients (24, 37.5%) compared to non-admitted patients (40, 16.1%, p < 0.001). Other locations did not differ significantly between groups (p > 0.05) (Table 3).

**Table 3:** Distribution of patients’ characteristics (N = 313) according to hospital admission ^†^ Some patients have multiple types and locations of injury. ^*^ Statistically significant at p < 0.05 level (chi-square test)

Characteristic	Hospital admission		p-value
	No (N = 249),	Yes (N = 64)	
Surgical treatment, N (%)			
Yes	3 (1.2)	33 (51.6)	<0.001^*^
No	246 (98.8)	31 (48.4)	
Injury type^†^, N (%)			
Abrasions	202 (81.1)	31 (48.4)	<0.001^*^
Lacerations	28 (11.2)	17 (26.6)	0.002^*^
Others	25 (10.0)	30 (46.9)	<0.001^*^
Injury location^†^, N (%)			
Head	40 (16.1)	24 (37.5)	<0.001^*^
Abdominal	11 (4.4)	5 (7.8)	0.271
Chest	17 (6.8)	5 (7.8)	0.783
Pelvic	21 (8.4)	6 (9.4)	0.811
Upper limb	132 (53.0)	32 (50.0)	0.667
Lower limb	166 (66.7)	39 (60.9)	0.390
Spine/back	11 (4.4%)	6 (9.4%)	0.119

Table 4 outlines the characteristics of patients based on trauma code activation. All patients in the activated group (25, 100%) were admitted, while only 39 patients (13.5%) from the non-activated group were admitted (p < 0.001). The activated group showed significantly higher rates of blood transfusion, with three patients (12.0%) compared to one patient (0.3%) in the non-activated group. CT scans were ordered for 19 patients (76.0%) in the activated group and 15 patients (5.2%) in the non-activated group. Endotracheal intubation was required for six patients (24.0%) in the activated group, with no cases in the non-activated group. These differences were statistically significant (all p < 0.001). Surgical intervention occurred in 13 patients (52.0%) in the activated group and 23 patients (8.0%) in the non-activated group (p < 0.001).

**Table 4:** Distribution of patients’ characteristics (N = 313) according to trauma code activation ^† ^Some patients have multiple types and locations of injury. ^*^ Statistically significant at p < 0.05 level (chi-square test) ^**^ Statistically significant at p < 0.05 level (Mann-Whitney U test)

Characteristic	Trauma code activation		p-value
	No (N = 288)	Yes (N = 25)	
Hospital admission, N (%)			
Yes	39 (13.5)	25 (100)	<0.001^*^
No	249 (86.5)	0 (0.0)	
Blood transfusion, N (%)			
Yes	1 (0.30)	3 (12.0)	<0.001^*^
No	287 (99.7)	22 (88.0)	
CT scan, N (%)			
Yes	15 (05.2)	19 (76.0)	<0.001^*^
No	273 (94.8)	06 (24.0)	
Endotracheal intubation, N (%)			
Yes	0 (0.0)	6 (24.0)	<0.001^*^
No	288 (100)	19 (76.0)	
Surgical treatment, N (%)			
Yes	23 (08.0)	13 (52.0)	<0.001^*^
No	265 (92.0)	12 (48.0)	
Injury type^†^, N (%)			
Abrasion	220 (76.4)	13 (52.0)	0.007^*^
Laceration	40 (13.9)	05 (20.0)	0.404
Others	42 (14.6)	13 (52.0)	<0.001^*^
Injury location^†^, N (%)			
Head	48 (16.7)	16 (64.0)	<0.001^*^
Abdomen	11 (03.8)	5 (20.0)	<0.001^*^
Chest	17 (05.9)	5 (20.0)	<0.001^*^
Pelvis	24 (08.3)	3 (12.0)	0.531
Upper limb	152 (52.8)	12 (48.0)	0.646
Lower limb	188 (65.3)	17 (68.0)	0.784
Spine/back	13 (4.5)	4 (16.0)	0.015^*^
Length of hospital stay in days, median (min-max)	1.00 (1.00-10.0)	1.00 (1.00-32.0)	<0.001^**^

Abrasions were less common in the activated group, reported in 13 patients (52.0%), compared to 220 patients (76.4%) in the non-activated group (p = 0.007). Other types of injuries were more frequent in the activated group, involving 13 patients (52.0%) compared to 42 patients (14.6%) in the non-activated group (p < 0.001). Head injuries were documented in 16 patients (64.0%) in the activated group and 48 patients (16.7%) in the non-activated group. Abdominal injuries affected five patients (20.0%) in the activated group and 11 patients (3.8%) in the non-activated group. Chest injuries were observed in five patients (20.0%) in the activated group and 17 patients (5.9%) in the non-activated group. These differences were also significant (all p < 0.001). Spine/back injuries were more common in the activated group, involving four patients (16.0%) compared to 13 patients (4.5%) in the non-activated group (p = 0.015). Other injury locations showed no significant differences (p > 0.05).

The median hospital stay was longer for the activated group, with a duration of one day (range 1-32), compared to one day (range 1-10) for the non-activated group (p < 0.001).

Findings in Table 5 indicate that trauma code activation was significantly associated with ambulance use, with 22 patients (12.6%) in the ambulance group compared to three patients (2.2%) in the non-ambulance group (p = 0.001), suggesting that severe cases warranting trauma activation were more likely to require ambulance transport. Regarding injury types, there were no significant differences between ambulance and non-ambulance groups for all types. For injury locations, head injuries were significantly more frequent among those transported by ambulance (43 patients, 24.6%) than those who were not (21 patients, 15.2%; p = 0.042). Pelvic injuries were also more common in the ambulance group (20 patients, 11.4%) compared to the non-ambulance group (seven patients, 5.1%; p = 0.047). However, no significant differences were observed for abdominal, chest, upper limb, lower limb, or spine/back injuries (all p >0.05).

**Table 5:** Relationship between ambulance use in terms of trauma code and injury type and location among the study patients (N = 313) ^† ^Some patients have multiple types and locations of injury. ^* ^Statistically significant at p < 0.05 level (chi-square test)

Characteristic	Ambulance used		p-value
	No (N = 138)	Yes (N = 175)	
Trauma code activation, N (%)			
Yes	3 (2.2)	22 (12.6)	0.001^*^
No	135 (97.8)	153 (87.4)	
Injury type^†^, N (%)			
Abrasions	101 (73.2)	132 (75.4)	0.652
Lacerations	17 (12.3)	28 (16.0)	0.357
Others	23 (16.7)	32 (18.3)	0.709
Injury location^†^, N (%)			
Head	21 (15.2)	43 (24.6)	0.042^*^
Abdomen	5 (3.6)	11 (6.3)	0.288
Chest	6 (4.3)	16 (9.1)	0.099
Pelvis	7 (5.1)	20 (11.4)	0.047^*^
Upper limb	70 (50.7)	94 (53.7)	0.599
Lower limb	91 (65.9)	114 (65.1)	0.883
Spine/back	06 (4.3)	11 (6.3)	0.453

Table 6 reveals significant associations between CT scan findings and specific injury types and locations. Injuries other than abrasions and lacerations were significantly more frequent in the CT group (N = 15, 44.1%) compared to the non-CT group (N = 40, 14.3%; p < 0.001). Head injuries were significantly higher among patients undergoing CT scans (N = 25, 73.5%, p < 0.001), as were spine/back injuries (N = 6, 17.6%; p = 0.001). No significant differences were observed for abrasions and lacerations or other locations. These results underscore the diagnostic emphasis of CT scans on head and spine/back injuries.

**Table 6:** Relationship between CT scan in terms of injury type and location among the study patients (N = 313) ^†^ Some patients have multiple types and locations of injury. ^*^ Statistically significant at p < 0.05 level (chi-square test)

Characteristic	CT scan		p-value
	No (N = 279)	Yes (N = 34)	
Injury type^†^, N (%)			
Abrasion	212 (76.0)	13 (38.2)	0.073
Laceration	38 (13.6)	7 (20.6)	0.274
Others	40 (14.3)	15 (44.1)	<0.001^*^
Injury location^†^, N (%)			
Head	39 (14.0)	25 (73.5)	<0.001^*^
Abdomen	12 (04.3)	4 (11.8)	0.062
Chest	17 (06.1)	5 (14.7)	0.064
Pelvis	24 (08.6)	3 (08.8)	0.965
Upper limb	146 (52.3)	18 (52.9)	0.946
Lower limb	186 (66.7)	19 (55.9)	0.212
Spine/back	11 (03.9)	6 (17.6)	0.001^*^

Table 7 displays significant associations between blood transfusion and certain injury patterns. Injuries classified as “others” were more frequent in patients who received blood transfusions (three patients, 75.0%) compared to those who did not (52 patients, 16.8%) (p = 0.002). Abdominal injuries were also strongly associated, occurring in two patients who received transfusions (50.0%) versus 14 patients who did not (4.5%) (p < 0.001). These findings suggest that blood transfusion is related to severe injuries, particularly abdominal injuries and less common injury types.

**Table 7:** Relationship between blood transfusion in terms of injury type and location among the study patients (N = 313) ^†^ Some patients have multiple types and locations of injury. ^*^ Statistically significant at p < 0.05 level (chi-square test)

Characteristic	Blood transfusion		p-value
	No (N = 309)	Yes (N = 4)	
Injury type^†^			
Abrasion	231 (74.8%)	2 (50.0)	0.271
Laceration	44 (14.2%)	1 (25.0)	0.464
Others	52 (16.8%)	3 (75.0)	0.002^*^
Injury location^†^			
Head	62 (20.1%)	2 (50.0)	0.187
Abdomen	14 (04.5%)	2 (50.0)	<0.001^*^
Chest	21 (06.8%)	1 (25.0)	0.254
Pelvis	26 (08.4%)	1 (25.0)	0.304
Upper limb	162 (52.4%)	2 (50.0)	1.000
Lower limb	202 (65.4%)	3 (75.0)	1.000
Spine/back	16 (05.2%)	1 (25.0)	0.201

Abrasions were significantly less frequent in patients undergoing surgical treatment (12 patients, 33.3%) compared to those who did not (221 patients, 79.8%; p < 0.001). Conversely, lacerations were more common in the surgical group (11 patients, 30.6%) compared to the non-surgical group (34 patients, 12.3%; p = 0.003). Several fractures were significantly associated with surgical treatment, including femoral fractures (five patients, 13.9%; p < 0.001), tibial fractures (eight patients, 22.2%; p < 0.001), fibula fractures (six patients, 16.7%; p < 0.001), ankle fractures (three patients, 8.3%; p = 0.001), spine fractures (three patients, 8.3%; p = 0.022), facial fractures (three patients, 8.3%; p = 0.001), clavicle fractures (four patients, 11.1%; p = 0.002), rib fractures (two patients, 5.6%; p = 0.036), pelvic fractures (two patients, 5.6%; p = 0.013), and skull fractures (three patients, 8.3%; p = 0.001). Other fractures, such as those involving the foot, radial, ulnar, olecranon, phalanx, orbital, metacarpal, and shoulder, showed no significant associations with surgical treatment.

For injury locations, head injuries were significantly more frequent in the surgical group (14 patients, 38.9%) compared to the non-surgical group (50 patients, 18.1%; p = 0.007). No significant differences were observed for abdominal, chest, pelvis, upper limb, lower limb, or spine/back injuries. These findings indicate that surgical treatment is often associated with more severe and specific fractures, particularly in the lower limbs, spine, and head (Table 8).

**Table 8:** Relationship between surgical treatment in terms of injury type and location among the study patients (N = 313) ^† ^Some patients have multiple types and locations of injury. ^* ^Statistically significant at p < 0.05 level (Fisher’s exact test)

Characteristics	Surgical treatment		p-value
	No (N = 277)	Yes (N = 36)	
Injury type^†^			
Abrasion	221 (79.8)	12 (33.3)	<0.001^*^
Laceration	34 (12.3)	11 (30.6)	0.003^*^
Femoral fracture	0 (0.0)	5 (13.9)	<0.001^*^
Tibial fracture	0 (0.0)	8 (22.2)	<0.001^*^
Fibula fracture	2 (0.70)	6 (16.7)	<0.001^*^
Ankle fracture	0 (0.0)	3 (8.3)	0.001^*^
Foot fracture	2 (0.70)	1 (2.8)	0.308
Spine fracture	3 (1.1)	3 (8.3)	0.022^*^
Scapular fracture	1 (0.40)	1 (2.8)	0.217
Humeral fracture	3 (1.1)	1 (02.8)	0.388
Radial fracture	5 (1.8)	1 (2.8)	0.523
Ulnar fracture	1 (0.40)	0 (0.0)	1.000
Olecranon fracture	1 (0.40)	0 (0.0)	1.000
Phalanx fracture	6 (2.2)	0 (0.0)	1.000
Orbital fracture	01 (0.40)	0 (0.0)	1.000
Facial fracture	0 (0.0)	3 (8.3)	0.001^*^
Clavicle fracture	2 (0.70)	4 (11.1)	0.002^*^
Ribs fracture	1 (0.40)	2 (5.6)	0.036^*^
Pelvic fracture	0 (0.0)	2 (5.6)	0.013^*^
Skull fracture	0 (0.0)	3 (8.3)	0.001^*^
Metacarpal fracture	4 (1.4)	0 (0.0)	1.000
Shoulder fracture	0 (0.0)	1 (2.8)	0.115
Injury location^†^			
Head	50 (18.1)	14 (38.9)	0.007^*^
Abdomen	12 (4.3)	4 (11.1)	0.098
Chest	17 (6.1)	5 (13.9)	0.154
Pelvis	25 (9.0)	2 (5.6)	0.752
Upper limb	145 (52.3)	19 (52.8)	1.000
Lower limb	181 (65.3)	24 (66.7)	1.000
Spine/back	13 (4.7)	4 (11.1)	0.117

## Discussion

Motorcyclists, especially delivery personnel, face significant accident risks due to unique work-related factors. This study examines motorbike accidents involving delivery personnel who were admitted to KHUH in the Kingdom of Bahrain. It highlights the occupational hazards they encounter, such as navigating urban traffic, tight delivery deadlines, and operating potentially poorly maintained bikes. The study sheds light on the risks these workers face, particularly those involving time pressures and frequent stops at busy locations.

Delivery personnel are more likely to be involved in accidents during peak hours, when time pressures and job-related distractions heighten the risk. In contrast, other riders are generally engaged in leisure or commuting activities, with a greater propensity for accidents due to speeding or reckless riding. However, systemic issues like insufficient training, limited safety awareness, and lack of regulation persist [9,10]. Comparing motorcycles to cars and trucks reveals that while larger vehicles offer greater protection and less risk, they also have their own set of risks, such as increased blind spots and longer stopping distances [11].

Most individuals involved in motorcycle accidents were under 30 years old, with an admission rate of 20.4%. The frequency of trauma activation codes, ambulance services, and medical interventions (including intubation, blood transfusion, CT scans, and surgery) varied. The most common injuries were to the lower limbs (65.5%), followed by the upper limbs (52.4%) and head (20.4%), consistent with studies emphasizing the vulnerability of extremities and the head in motorcycle accidents [12-14]. Regional studies show variations influenced by local factors and riding behaviors [15-20]. For example, Hsieh et al.’s study on older individuals revealed different injury patterns and higher mortality rates, particularly in head injuries [21].

The injury pattern in this study aligns with trends observed in other motorbike-related occupations, with lower limbs most frequently affected, followed by upper limbs and head injuries. This pattern reflects the mechanics of motorbike accidents, where the lower body is often exposed to impact, and riders are prone to falling or being ejected during crashes. Our study found a high rate of lower-limb injuries (65.5%), likely due to rider posture and leg vulnerability. In contrast, studies from other regions show some variation in injury patterns. For instance, a study in the UAE found that upper limbs were most commonly injured (54%), followed by lower limbs (48%), head (41%), and face (30%) [22]. Similarly, research in Iran indicated that upper and lower limb trauma occurred in 78.8% and 60.9% of cases, respectively [23]. Upper limb injuries, often seen in over 50% of cases, are typically associated with reflexive actions as riders attempt to break their fall or shield themselves. These findings highlight the combined role of anatomical exposure and protective reflexes in the distribution of injuries in motorbike-related incidents.

Abrasions (74.4%) and lacerations (14.4%) were the most frequent injuries, indicating that delivery personnel are often involved in less severe but still debilitating accidents. Abrasions are the most common injury in motorcyclists, even with full protective gear [24,25]. More severe injuries, like fractures, were less frequent in our study. These injuries, often resulting from high-impact incidents, are linked to higher risks of long-term disability. Although fractures (such as shoulder, orbital, olecranon, and ulnar fractures) were less frequent in our study, their lower incidence likely reflects the protective effects of helmets and other safety equipment.

Delivery personnel face specific risks that other motorcyclists do not, including time-sensitive traffic hazards and potential conflicts with merchants and customers. The income dependence of these riders has been positively associated with increased work-related injuries, with workload serving as a mediator [26].

Our study found that trauma code activation was associated with higher hospital admission rates, highlighting the importance of prompt trauma care, as seen in other global data [14,27-29]. The increased need for trauma code activation corresponded with cases requiring blood transfusions, CT scans, intubation, and surgical intervention, underlining the crucial role of trauma teams in managing complex injuries.

In comparison to the Guinea study, which reported motorcycle RTA accounting for 58.3% of hospital admissions, Bahrain’s rate was 20.4% [20]. Other studies also report a pediatric motorcycle-related trauma admission rate of 22.2 per 100,000 population [30]. Our study did not identify other studies linking trauma code activation to hospital admissions and blood transfusions.

Additionally, we observed that ambulance use was significantly associated with trauma code activation, which underscores the importance of timely transport to specialized care. Ambulances were notably more used in cases involving head and pelvic injuries, emphasizing the need for rapid transport for these high-risk injuries [27,31,32].

The need for blood transfusions was significantly higher in patients with abdominal and other severe injuries (p = 0.002), indicating that these injuries often result in more severe bleeding. We also observed that patients with head injuries, spine/back trauma, and other serious injuries (p < 0.001) were more likely to undergo CT scans. This highlights the need for detailed imaging, especially for head and spine injuries. This practice aligns with findings from a German study, where whole-body CT scans were increasingly used for effective diagnosis [33]. Most patients (88.5%) in our study did not require surgical procedures, with a median hospital stay of just 1.0 days. Surgical procedures were more common in cases with specific injuries like lacerations and fractures (femoral, tibial, fibula, ankle, facial, clavicle, ribs, and skull fractures), while abrasions, typically less severe, were less likely to require surgery.

In a related study focusing on elderly individuals hospitalized due to trauma from motorcycle accidents, the researchers found that this demographic experienced higher injury severity, particularly in relation to helmet usage. Those without helmets had a higher proportion requiring ICU admission and surgery, highlighting the critical role of surgical interventions, particularly for head injuries [21]. Similarly, in the following study, the specific surgical interventions, although not detailed, suggest a correlation between head injuries, common outcomes of motorcycle accidents, and the potential need for urgent medical interventions, including surgical procedures [27]. These findings reinforce the importance of protective measures like helmets and proper gear in mitigating injury severity, particularly head injuries.

Safety compliance in the delivery sector includes safe riding, using personal protective equipment, vehicle maintenance, and adhering to traffic regulations. Riders who prioritize safety are more likely to follow these practices, reducing accident risks [34]. The CDC has highlighted universal helmet laws as the most effective strategy to reduce motorcyclist deaths and associated costs [35,36]. Wearing helmets can reduce the risk of fatality and head injuries by approximately 42% and 69%, respectively [37]. Despite the availability of helmets, compliance remains low, particularly in East Asia, where helmet usage rates can be as low as 3% [38-40]. Even when helmets are available, discomfort, lack of employer enforcement, and limited awareness of their importance contribute to low usage. The quality of helmets plays a crucial role in the severity of head, neck, and facial injuries, and substandard helmets may fail to provide adequate protection, resulting in more severe outcomes. Additionally, the effectiveness of other protective gear like gloves, elbow and knee guards, and reinforced jackets is less well understood, although these have been associated with reduced injury severity and hospitalizations when used with body armor [41]. Employers can help mitigate risks by enforcing safety gear policies, providing high-quality equipment, and educating riders on its importance.

Our study found that motorcycle incidents peaked in 2017 and were lowest in 2019, affecting primarily younger males. In comparison, the Guinea study reported consistently high motorcycle-related RTAs and mortality from 2015 to 2017, with some decrease over time, although certain groups remained disproportionately affected [20]. Similarly, the study in Germany showed improvements in injury severity and the use of whole-body CT scans, alongside a decline in the hospital mortality rate for polytraumatized motorcyclists [33]. A study examining aggressive driving among on-demand food delivery motorcyclists found a correlation between aggression levels and meal-peak hours, suggesting that factors like pay rate surges may influence driving behavior [4]. A study in the UAE on speeding incidents noted a decline after the implementation of radar systems and speed cameras [42].

Our study aligns with global concerns about motorcycle accidents, especially among delivery personnel, echoing the need for a concerted effort to address this public health challenge [31,43,44]. The observed trends in Bahrain share similarities with global reports, emphasizing the widespread nature of the issue.

Despite its value, this study has limitations. The retrospective nature of the data from 2016 to 2019 may introduce biases and limit causal conclusions. The study's focus on a single region and center may restrict the applicability of the findings to other populations. Furthermore, the accuracy of medical records may affect the completeness of the data, and temporal changes such as advances in medical care or changes in traffic regulations may influence the relevance of the findings. Acknowledging these limitations is essential for interpreting the results and guiding future research on motorcycle accidents among delivery personnel.

## Conclusions

This study highlights the risks faced by young delivery personnel in Bahrain, with lower limb injuries and head trauma being prevalent. Despite the effectiveness of current trauma care protocols in managing injuries, gaps remain in preventing severe injuries. The research emphasizes the need for region-specific measures, such as mandatory helmet laws, the use of protective gear, and the establishment of safer routes for delivery personnel. Furthermore, local awareness campaigns about safe riding practices and enhanced training programs for delivery staff can play a crucial role in reducing injury risks. By providing unique data on injury patterns and trauma care in the Gulf region, the study contributes to improving road safety and trauma management while supporting further research and policy development tailored to the specific needs of delivery personnel in Bahrain.
